# Biomechanical Monitoring of Exercise Fatigue Using Wearable Devices: A Review

**DOI:** 10.3390/bioengineering13010013

**Published:** 2025-12-24

**Authors:** Yang Chen, Siqi Li, Jian Kuang, Xu Zhang, Zhijie Zhou, En-Jing Li, Xiaoli Chen, Xianmei Meng

**Affiliations:** 1 School of Nursing, Wuhan University, Wuchang District, Wuhan 430072, China; mynamechenyang@outlook.com (Y.C.); m18272000883@163.com (S.L.); wszhangxu2006@whu.edu.cn (X.Z.); zouzhijie@whu.edu.cn (Z.Z.); 2 GNSS Research Center, Wuhan University, Wuchang District, Wuhan 430072, China; kuang@whu.edu.cn; 3 Physical Culture Institute, Central China Normal University, Hongshan District, Wuhan 430079, China

**Keywords:** wearable, exercise fatigue, biomechanical, fatigue diagnosis, real-time monitoring, injury prevention

## Abstract

Exercise fatigue is a critical factor that compromises athletic performance, increases the risk of musculoskeletal injury, and threatens safety in military and occupational settings. Reliable monitoring of fatigue is therefore essential for optimizing training, preventing injury, and safeguarding long-term health. Biomechanical indicators, including joint kinematics, ground reaction forces, and electromyographic signals, provide valuable insight into the biomechanical manifestations of fatigue. Although traditional laboratory-based methods are accurate, they are costly, cumbersome, and unsuitable for continuous field monitoring. Recent advances in wearable technologies, particularly inertial measurement units (IMUs), insole pressure sensors (IPSs), and surface electromyography (sEMG), enable continuous, noninvasive, and real-time assessment of biomechanical changes during exercise fatigue. This review synthesizes current progress in IMU-, IPS-, and sEMG-based wearable systems for biomechanical exercise fatigue monitoring, highlighting their principles, strengths, and challenges.

## 1. Introduction

Exercise fatigue is a condition in which the body is unable to maintain an activity at a consistent level or to sustain a predetermined level of intensity during physical activity [1]. Exercise fatigue is a pervasive challenge across sports, occupational health, and military training, where it compromises performance, raises injury risk, and affects overall safety [2]. According to an epidemiological survey conducted in China in 2020, excessive fatigue and acute injury are the main causes of military training injuries, with an incidence rate of 10–20%, which is much higher than the 8% stipulated in the “Military Training Injury Health Protection Regulations” [3]. According to a survey in the United States in 2019, the death toll resulting from military accidents caused by excessive fatigue accounts for one-third of the annual death toll in the United States military [4]. The physical exhaustion and fatigue experienced by soldiers due to rigorous and prolonged training can have a detrimental effect on their military operations and cognitive abilities [5]. Athletes who experience fatigue during exercise may struggle to maintain normal performance and are vulnerable to injury [6]. Beyond immediate effects, cumulative fatigue can contribute to chronic overuse syndromes and compromise long-term well-being. Given its widespread impact, reliable monitoring of exercise fatigue is essential not only for safeguarding health but also for optimizing performance and preventing injuries [7].

Exercise fatigue is a complex, multifactorial process encompassing both central mechanisms and peripheral mechanisms, but it can be broadly understood as a comprehensive reaction of various physiological and biochemical changes in the body [8,9]. These physiological processes manifest biomechanically as alterations in movement patterns, joint loading, and neuromuscular activity [10]. Indicators such as joint kinematics, ground reaction forces, and electromyographic signals provide critical insights into how fatigue reshapes movement strategies and compromises stability. Therefore, accurate detection of these biomechanical signatures is essential for understanding fatigue progression and designing timely interventions. In previous studies, traditional biomechanical assessments typically relied on laboratory-based systems, including optical motion capture, force platforms, and wired electromyography (EMG) setups [11]. While these tools offer high precision, they are constrained by high costs, complex instrumentation, environmental requirements, and the need for trained operators. Their lack of portability limits ecological validity, making them unsuitable for continuous monitoring in training fields, clinical rehabilitation, or occupational environments.

The integration of wearable technologies with biomechanical analysis for exercise fatigue monitoring has gained significant traction in recent years. Wearable fatigue-monitoring prototypes have evolved to be deployable across diverse settings, including training fields, clinical environments, and occupational workplaces [12,13,14]. Devices incorporating inertial measurement units (IMUs), insole pressure sensors (IPSs), and surface EMG electrodes allow for continuous, non-invasive assessment of fatigue-related biomechanical changes during exercise [15]. Recent advances in machine learning, deep learning architectures, and multi-sensor fusion techniques have further expanded the analytical capacity of wearable systems, enabling higher precision fatigue-state recognition and monitoring.

As research on exercise fatigue progresses, many scholars have begun to develop and apply wearable devices in military training and exercise monitoring. However, few have focused on the biomechanical changes associated with exercise fatigue or integrated insights from IMU-, IPS-, and EMG-based approaches [16,17,18]. Despite significant advancements in these sensing technologies, their specific potential for analyzing the biomechanics of exercise fatigue remains underexplored. A comprehensive review focusing on wearable device-based biomechanical monitoring is therefore warranted to consolidate existing knowledge, identify critical research gaps, and outline pathways for future innovation.

In this paper, exercise fatigue refers to task-specific fatigue induced by physical activity, excluding chronic or pathological forms of fatigue as well as cognitive or psychological fatigue [19]. The scope of this review is restricted to peripherally manifested, exercise-induced fatigue that can be captured through biomechanical monitoring systems. First, we summarize the biomechanical indicators that are most relevant for fatigue detection, including joint kinematics, ground reaction forces, and neuromuscular activities. Next, we examine advances in wearable monitoring technologies across IMU, IPS, and EMG modalities, highlighting their principles, applications, and challenges. Finally, emerging opportunities of wearable biomechanical sensors are prospected. By synthesizing current evidence and outlining future directions, this work seeks to inform the development of robust, real-world wearable systems capable of transforming fatigue monitoring across sports and occupational health domains.

## 2. Biomechanical Indexes for Wearable Devices and Their Application in Exercise Fatigue Monitoring

Exercise fatigue induces notable changes in biomechanical indexes, such as kinematic parameters, ground reaction force, myoelectric activity, and other indicators, offering insight into the biomechanical effects of fatigue on the body [10]. In a non-invasive way, many wearable devices have been developed in recent years to monitor these biomechanical indicators (Table 1).

### 2.1. Biomechanical Monitoring Based on IMUs

#### 2.1.1. Kinematic Parameters in IMU Monitoring

IMUs are wearable sensing units that typically integrate tri-axial accelerometers and gyroscopes, and in some cases magnetometers, to capture segment-level linear acceleration and angular velocity in three dimensions [51,52]. In biomechanical fatigue monitoring, IMUs are firmly attached to relevant body segments (e.g., shank, thigh, trunk). During human movement, IMUs fixed on specific anatomical locations continuously capture raw acceleration and angular velocity signals associated with segment motion. These signals are first pre-processed to reduce noise and motion artifacts, and then estimate sensor orientation in a global reference frame [53] by using sensor fusion algorithms, such as complementary filters or extended Kalman filters. After reconstructing the orientations of adjacent body segments, the relative joint motion can be quantified in real time, yielding key kinematic parameters [53]. The processed kinematic data are subsequently transmitted wirelessly to an external device for further analysis, enabling continuous and high-resolution monitoring of human movement under both laboratory and real-world conditions.

**Figure 1:**
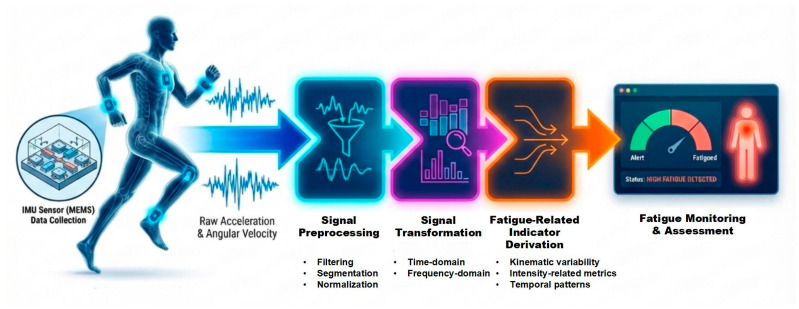
Overview of an IMU-based signal processing pipeline for exercise fatigue monitoring.

Figure 1 summarizes key stages from data acquisition and preprocessing to signal transformation and fatigue-related indicator derivation, leading to fatigue assessment. It represents a generalized methodological framework and does not correspond to any specific machine learning implementation or network structure.

Compared with other common motion tracking systems, such as mechanical, optical, acoustic, and electromagnetic systems [54], IMU-based systems provide a noninvasive approach to capturing fatigue-related changes in body movement and posture [30] with independence from line-of-sight constraints and external laboratory infrastructure. They are fully wearable, lightweight and low-cost, enabling continuous monitoring in real-world environments such as outdoor running tracks, construction sites or rehabilitation settings. Although a variety of skin-integrated flexible strain sensors have been developed in recent years for joint motion monitoring, their performance is still limited by environmental stability and nonlinear response [55,56]. In contrast, IMUs are largely insensitive to variations in temperature, humidity, and sweat interference [57]. The signals captured by IMUs show a closer linear correspondence to true segmental motion, with superior sensor calibratability and improved generalizability across individuals [58].

Exercise fatigue is mainly reflected in the biomechanical changes of the lower extremity, especially changes in joint kinematics [59], such as joint angle and joint torque. At present, studies on changes in the joint angle under exercise fatigue are primarily carried out from the sagittal and frontal planes of the human body. From the sagittal perspective of biomechanics, when the body enters the state of exercise fatigue, the hip joint, knee joint, and ankle joint of the exerciser show significant angle changes in different stages of body fatigue. With the development of the fatigue process, the maximum flexion angle of the hip joint increased significantly [60]. However, for the knee joint, different or even contradictory results have been found regarding the change in the peak flexion angle. Luo et al. [61] reported a significant increase in knee flexion angle after fatigue. However, Patrek et al. [62] found that the peak value of the knee flexion angle did not change after fatigue. Chappell et al. [63] found that the peak value of knee flexion angle decreased after fatigue. Kernozek et al. [64] reported that the peak value of the knee flexion angle increased from the occurrence of fatigue to the middle stage of fatigue, but no significant change was found in the later stage of fatigue. Abt et al. [65] observed that participants had a larger knee flexion angle in the later stage of fatigue. When discussing the effects of fatigue on the ankle joint, the results have been consistent, and the angle of dorsiflexion of the ankle decreases during the whole process of fatigue [66,67]. In addition, Zhang et al. [68] found that the peak flexion angles of the hip, knee and ankle increased during moderate fatigue, while they decreased during severe fatigue. In the frontal view of biomechanics, the abduction angle of the hip joint (i.e., the peak moment of knee flexion) and the adduction angle of the knee joint increase due to the emergence of the exercise fatigue state. Changes in the angle of the hip and knee joints affect the stability of the human body and increase the risk of bodily injuries. One study exploring exercise fatigue among female football players found that the abduction angle of the hip joint and the internal rotation angle of the knee joint increased compared to those before fatigue [69]. Dickin et al. [70] also determined that under fatigue, abduction of the hip joint and the valgus angle of the knee joint increased.

After strenuous or long-term exercise, fatigue can result in a decrease in muscle strength and control, which impairs the body’s ability to generate the force necessary for joint movement. This impairment subsequently influences variations in joint torque. One study of jump-induced fatigue explored that muscle contraction and coordination capacities declined under fatigued conditions, which may lead to a decrease in extension torque of the hip and knee joints [71]. Similarly, Kernozek et al. [64] found that the torque of the hip joint decreased by about 25% in the early stage of fatigue. In contrast, Murdock et al. [72] showed that an increase in the valgus moment of the knee joint during exhaustion or near exhaustion may be due to a decreased ability of the musculoskeletal system to maintain joint stability in walking-induced fatigue. Ankle joint torque exhibits a more consistent downward trend after fatigue. Jayalath et al. [73] found that ankle torque decreased to 10–20% of the standing value following jump-induced fatigue, while Schmitz et al. [74] observed similar reductions after running-induced fatigue.

#### 2.1.2. Application of the IMU

**Figure 2:**
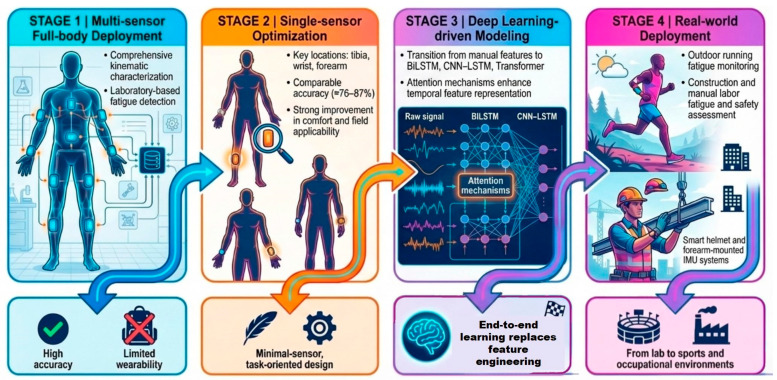
IMU-based Fatigue Monitoring: Technology Trends.

In recent years, fatigue monitoring based on IMU has been extensively investigated in both sports and occupational settings, with each domain emphasizing practical challenges (Figure 2). Recent studies have demonstrated that IMU is capable of capturing fatigue-related biomechanical alterations when placed on the shank, thigh, pelvis, or trunk, including changes in stride frequency, stride variability, angular velocity, and impact accelerations [26,27,28,29].

In the field of sports training, IMU is primarily applied in fatigue monitoring of lower extremity-dominated sports such as running. Op De Beéck et al. [23] positioned multiple IMUs on the wrists, arms, and tibias of 8 runners, employing data-driven methods to predict fatigue under outdoor conditions. The study found that the single wrist sensor provided the most accurate prediction (mean absolute error of 1.89), while adding multiple sensors yielded only marginal performance gains. Marotta et al. [26] evaluated the influence of different IMU placements on detection accuracy in outdoor running fatigue, finding that a single sensor on the left tibia achieved 76% accuracy, whereas a full-body multi-sensor configuration reached 90.5%. Similarly, Wang et al. [29] used lower-limb IMU to identify and classify fatigue in laboratory settings, reporting that a single tibial sensor achieved a classification accuracy of 87%, which increased to 91% when combined with a thigh-mounted sensor. The study also emphasized that inter-individual variability and environmental noise remain significant challenges to model stability. Overall, these findings highlight the effectiveness and reliability of IMU-based systems for fatigue monitoring in sports contexts.

In occupational settings, IMU-based fatigue monitoring among construction and manual labor workers primarily focuses on safety alerts and real-time workload assessment. Li et al. [21] investigated a Smart Safety Helmet (SSH) equipped with IMU and EEG sensors to detect fatigue-related head movements and assess accident risks, utilizing precise acceleration and orientation measurements for fatigue identification. Bangaru et al. [75] introduced an IMU-based system integrating forearm-mounted sensors with recurrent neural networks to achieve continuous monitoring of construction workers’ fatigue, demonstrating superior accuracy compared with traditional indicators. In subsequent research, the same group employed a BiLSTM model using forearm-derived IMU features to estimate construction workers’ fatigue status and oxygen consumption, reporting strong agreement with gold-standard measurements (R = 0.90) [31]. Additionally, Bonakdar et al. [76] employed wearable IMUs to monitor joint-angle variations during multi-task manual labor, identifying fatigue-induced declines in coordination and movement stability. Although the CNN-LSTM model used in this study achieved only 77% accuracy in multi-level fatigue classification, it still delivered competitive performance with minimal data and computational resources, offering a more practical solution for real-time fatigue monitoring in industrial environments.

With advancements in data analysis technologies, a growing number of studies have incorporated machine learning and deep learning algorithms to reduce the burden of manual feature extraction and improve fatigue-classification performance and recognition accuracy. Guan et al. [24] combined ECG signals with IMU and employed a BiLSTM network to classify three fatigue levels, achieving an accuracy of 80.55%. Jiang et al. [27] further advanced the field by developing a Transformer-based model capable of real-time fatigue recognition and forecasting future fatigue progression, achieving a predictive accuracy of 83.1%. Chang et al. [30] demonstrated that the incorporation of an attention mechanism enhances the feature-extraction capability of LSTM models applied to raw IMU data. Furthermore, the hybrid architecture combining CNN and LSTM outperforms a single model in fatigue classification tasks, achieving accuracy rate of 86.3%. In recent work, Hwang et al. [32] proposed a CNN-LSTM-Attention hybrid model that integrated sEMG and IMU signals, achieving 87.94% accuracy in cross-subject validation.

### 2.2. Biomechanical Monitoring Based on the IPS

#### 2.2.1. Kinematic Parameters of IPS Monitoring

Exercise fatigue induces a series of biomechanical alterations that can be captured through plantar pressure signals, including ground reaction force, plantar pressure distribution, peak pressure, and contact duration. In biomechanical research, the ground reaction force is the opposite force exerted by the ground on an object, and it is a commonly used sensitive index to reflect the ability of the musculoskeletal system to withstand external loads [77]. During the gradual development of the body’s fatigue state, the ground reaction force received by the human body will continue to increase, and the pulling force received by the relevant muscle group will also increase [78]. In addition, a common outcome of fatigue is the redistribution of loading among distinct plantar regions [79]. During fatigue, there is a significant decrease in phalangeal pressure, coupled with a notable increase in metatarsal pressure [79]. Simultaneously, the contact time between the phalanges and the ground decreases significantly, whereas the contact time between the metatarsal and heel and the ground increases significantly [79]. Evidence from a running fatigue experiment indicated that prolonged or high-intensity running markedly elevated plantar peak pressure and caused redistribution of plantar loads [36]. Post-fatigue pressure alterations varied among runners depending on their foot-strike type: rearfoot strikers exhibited increased pressure in the medial and lateral heel regions, as well as the first metatarsal and hallux areas following fatigue; whereas forefoot strikers primarily demonstrated elevated pressure from the first to third metatarsals [80]. These variations in distribution offer a direct approach to quantifying fatigue.

Based on sensing mechanisms, IPS can be classified into four types: photoelectric sensors, force-sensitive resistors, capacitive sensors, and piezoelectric sensors [81]. The first three types of sensors depend on an external power supply, whereas self-powered piezoelectric sensors can efficiently convert pressure into electrical signals. It offers the unique advantage of non-invasive, real-time monitoring of plantar pressure changes indicative of lower-limb fatigue, and can be seamlessly incorporated into daily footwear [82]. Wearable IPS systems operate by embedding distributed pressure-sensitive elements within a flexible insole to continuously capture plantar load variations during locomotion. The sensed mechanical forces are transduced into electrical signals through resistive, capacitive, or piezoelectric mechanisms, followed by signal conditioning, analog-to-digital conversion, and wireless transmission. Spatiotemporal plantar pressure features are subsequently extracted to quantify fatigue-related changes in gait mechanics and plantar loading patterns [82]. These features are further mapped to fatigue states using statistical analysis or machine learning models, thereby enabling real-time fatigue monitoring and feedback in unconstrained environments. IPS directly captures fatigue-related alterations in foot–ground interaction that cannot be observed by kinematic or neuromuscular sensing modalities, thereby providing a distinct biomechanical perspective on fatigue progression.

**Figure 3:**
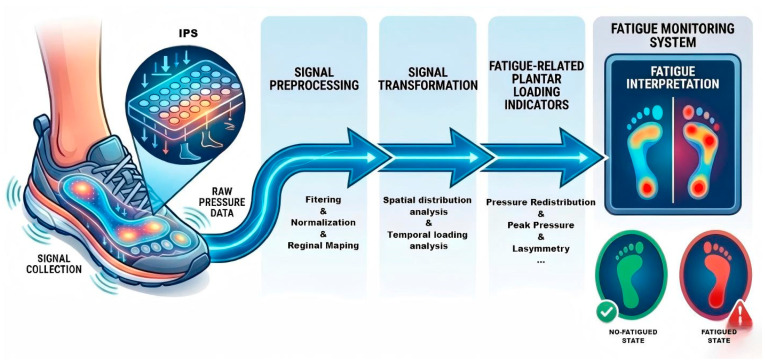
Overview of an IPS-based signal processing pipeline for exercise fatigue monitoring.

Figure 3 illustrates a conceptual workflow for IPS-based fatigue monitoring, highlighting key signal processing and feature derivation stages rather than specifying any particular machine learning architecture.

#### 2.2.2. Application of the IPS

**Figure 4:**
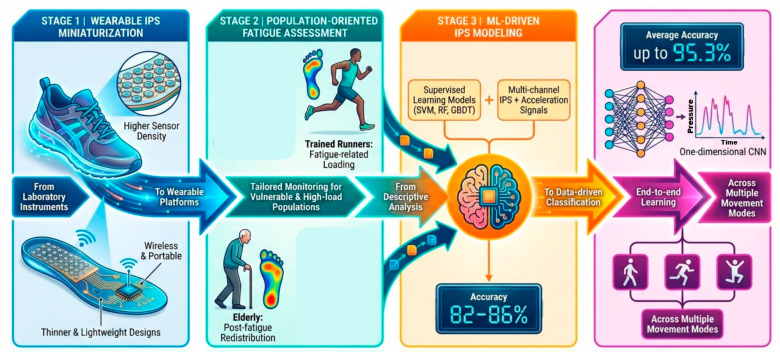
IPS-based fatigue monitoring: technology trends.

In contrast to conventional pressure plates or laboratory testing platforms, modern IPS platforms are typically wireless and portable, with increased sensor density, higher sampling frequencies, and thinner, lightweight designs that minimize user discomfort (Figure 4).

Recently, wearable IPS technology has gained increasing research attention for its potential to enable real-time fatigue assessment in specific populations, particularly the elderly and athletes. Using the Novel Pedar-X in-shoe pressure mapping system, Zhang et al. [34] examined plantar pressures in older adults following extended fast walking, observing elevated peak pressures in the arch and metatarsal regions as well as prolonged contact durations. These gait feature changes suggest a decline in cushioning capacity associated with fatigue, as well as a potential compensatory strategy in older adults, in which stability is preserved by extending ground contact duration. Although this assumption still requires additional empirical evidence, it underscores the need to integrate personalized adaptation strategies into fatigue monitoring for elderly individuals. Buxton et al. [36] extended the population to trained runners and evaluated plantar peak pressures across speeds ranging from walking to sprinting. This work successfully captured post-fatigue alterations in plantar loading across multiple speed conditions, providing preliminary evidence supporting the application of IPS for near-real-world fatigue monitoring.

Based on these results, investigators have sought to capture fatigue features more reliably and accurately in exercise or high-load scenarios. Consequently, subsequent studies have incorporated supervised learning techniques (e.g., SVM, RF, GBDT) to enhance the sensitivity of fatigue recognition. An eight-channel fatigue recognition system integrating plantar pressure with acceleration signals was developed by Cao, utilizing RF, SVM, and GBDT methods [75]. The findings showed that random forest attained 82.5% accuracy, surpassing competing models. In a study simulating construction work, Antwi-Afari et al. [35] combined IPS and acceleration with RF and SVM to categorize three fatigue intensities, with RF achieving the highest accuracy (86%). This study demonstrated the potential of IPS in high-load working environments; however, its generalizability is constrained by the small sample size (n = 10). As research progressed, the Tian group developed a one-dimensional convolutional neural network (1D-CNN) capable of extracting fatigue features directly from plantar-pressure time series collected during walking, running, and jumping. The model enabled accurate fatigue monitoring across multiple movement modes, achieving an average recognition accuracy of 95.3% and substantially outperforming traditional machine-learning models. The capacitive-array wearable insole system is capable of real-time plantar pressure mapping and fatigue detection, while may further extend to podiatric prevention and evaluation of injury risks.

It should be noted that, whether in laboratory or real-world settings, IPS systems are limited to capturing plantar-pressure information only during the stance phase and cannot acquire pressure data during the swing phase [83]. Consequently, when the research objective is to quantify cumulative fatigue across complete gait cycles, IPS needs to be integrated with complementary sensing modalities such as IMU and sEMG. To address this shortcoming, Zhang et al. [84] developed a smart portable system combining IMU and IPS for the automated detection, achieving 99% detection accuracy, markedly higher than the 95% obtained using IPS alone. In contrast, Chen et al. [85] developed a digital insole platform that combines plantar pressure with gastrocnemius sEMG frequency-domain features, thereby enhancing the robustness of IPS-based fatigue assessment. The study also highlighted the potential of IPS for home-based monitoring and recommended future integration with mobile applications to enable continuous, user-friendly fatigue surveillance.

### 2.3. Biomechanical Monitoring Based on sEMG Sensors

#### 2.3.1. Kinematic Parameters in sEMG Sensor Monitoring

**Figure 5:**
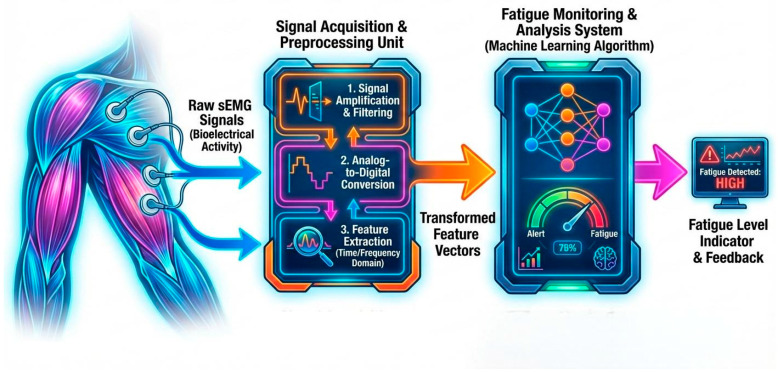
Process of collecting and transforming signals to monitoring exercise fatigue using sEMG.

Wearable sEMG systems typically consist of pairs or arrays of surface electrodes, signal amplification and conditioning circuits, and wireless transmission modules for recording muscle electrical activity in a non-invasive manner [86]. In biomechanical fatigue monitoring, sEMG electrodes are attached to the skin surface over target muscles (e.g., quadriceps, hamstrings, gastrocnemius, or tibialis anterior). During muscle contraction, motor unit action potentials are generated and propagate along muscle fibers, giving rise to time-varying bioelectrical potentials at the skin surface. These raw sEMG signals are continuously acquired by surface electrodes, amplified, and band-pass filtered to suppress motion artifacts and power-line interference. The processed signals are then converted into digital form and segmented into analysis windows, from which fatigue-related features are extracted. These features are subsequently used by machine learning algorithms or conventional classifiers to quantify muscle activation patterns and fatigue states in real time [86]. The processed sEMG data can be transmitted wirelessly to external devices for visualization and further analysis, enabling continuous monitoring of neuromuscular function under both laboratory and real-world conditions (Figure 5).

Wearable sEMG systems provide a non-invasive yet practical approach for real-time monitoring of muscle function, which is particularly valuable for identifying overuse of specific muscle groups during sports training, rehabilitation exercises, and occupational tasks [87]. sEMG is highly sensitive to early fatigue-induced changes in motor unit recruitment strategies and muscle fiber conduction velocity, allowing fatigue to be detected even before pronounced kinematic alterations become evident. Additionally, sEMG can be used with other physiological measures, such as heart rate variability or electroencephalography, to provide a more complete picture of fatigue. These innovations contribute to a better understanding of muscle physiology and offer practical solutions to preventing fatigue-related injuries and optimizing performance in various settings [88].

Currently, sEMG sensors are employed to assess the degree of exercise fatigue through continuous tracking of time-domain, frequency-domain, and nonlinear parameters of electromyographic signals. Amplitude-based indicators of EMG typically exhibit an upward trend as muscle fatigue intensifies. Typical time-domain parameters, including the root mean square (RMS) and integrated EMG (iEMG), tend to rise as fatigue deepens, thereby indicating increased contractile force and higher energy consumption [89,90]. Frequency-domain features such as mean frequency (MF) and median frequency (MDF) reflect changes in muscle fiber conduction properties and metabolic status. Under fatigue conditions, both MF and MDF exhibit a linear decline over time, with the rate of frequency reduction accelerating as fatigue deepens [91,92]. Furthermore, the decrease in muscle fiber conduction velocity has been recognized as one of the critical parameters for the quantitative assessment of muscle fatigue [86]. In addition to the traditional time- and frequency-domain indices, researchers have increasingly employed nonlinear measures, such as approximate entropy and sample entropy, to capture fatigue signatures, offering a more holistic representation of the dynamic changes in sEMG signals during fatigue development [93,94,95,96,97].

#### 2.3.2. Application of sEMG Sensors

**Figure 6:**
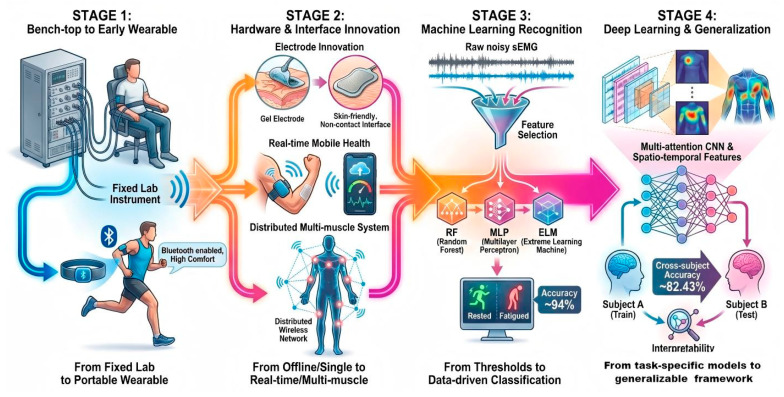
sEMG-based Fatigue Monitoring: Technology Trends.

In recent years, the emergence of various wearable sEMG sensors makes continuous monitoring of muscle fatigue during exercise feasible (Figure 6). Initial investigations concentrated on designing wearable sEMG devices to facilitate portable fatigue monitoring and reliable detection. Heaffey et al. [40] designed one of the first wearable sEMG monitoring devices, which maintained measurement accuracy equivalent to traditional bench-top systems under Bluetooth transmission, while substantially improving freedom of movement and wearing comfort. Subsequently, Kobayashi et al. [41] developed a wearable system employing capacitive-coupled electrodes to overcome the limitations of traditional Ag/AgCl electrodes, such as gel dependency, dehydration, and skin irritation. This device not only minimized skin irritation and dependence on consumables but also demonstrated feasibility for daily fatigue monitoring in amateur soccer players. Moreover, the commercially available and low-cost Myo armband has also been employed for fatigue detection during running and upper-limb tasks [42]. To further enable real-time fatigue monitoring in practical scenarios, Liu et al. [43] developed a wearable “smart patch” affixed to the gastrocnemius, which computes MF in real time during exercise and transmits results to a mobile application. In tests with 20 cycling volunteers, the patch-recorded MF deviated from laboratory offline calculations by merely 2–3 Hz, confirming the accuracy and feasibility of continuous fatigue monitoring using wearable patches. Based on these advancements, Song et al. [46] introduced a wireless distributed approach using miniature sensors to simultaneously monitor multiple muscles, while wireless charging and miniaturization enhanced deployment flexibility. Moreover, Gong et al. [48] developed a flexible wireless sEMG system that used gold-based flexible electrodes and stretchable circuitry to ensure secure attachment, combining multi-domain features for intelligent fatigue recognition. In addition, Gehlot et al. [47] developed a wearable sEMG system based on IoT technology, employing time–frequency domain analysis to classify three levels of muscle fatigue: relaxed, moderate, and extreme fatigue. The system presents different fatigue states through a LabVIEW interface and LED alarms, and uses a built-in Wi-Fi module for cloud-based log recording.

As data availability and computational power have increased, the field of EMG-based fatigue recognition has shifted from simplistic, low-adaptability thresholding methods toward data-driven machine learning and deep learning approaches, which aim to achieve higher accuracy and improved generalization across populations. Conventional machine learning methods achieved high accuracy by exploiting data-driven pattern recognition. Khan et al. [44] applied a random forest model with feature selection, maintaining high accuracy while reducing model complexity. Bharathi et al. contrasted multilayer perceptron (MLP) with extreme learning machine (ELM) models and reported an accuracy of 94% [98]. Although these approaches yielded favorable results in individual datasets, they were largely tailored to particular muscles and tasks, thereby limiting their broader applicability across diverse populations. To address challenges of cross-subject variability, Zhang et al. [50] introduced the multi-attention convolutional network (MACNet), a deep learning architecture designed to capture both temporal and spatial EMG features, enabling multi-grade evaluation of exercise fatigue. MACNet achieved a cross-subject accuracy of 82.43%, significantly outperforming conventional classifiers including SVM and RF. Importantly, the researchers employed analysis of the deep model, revealing that muscle channels like the forearm flexor digitorum superficialis contributed substantially to high fatigue levels, and that temporal segments differed in their influence on model decisions [50]. This not only validated the effectiveness of deep learning models in extracting fatigue-related information but also identified key EMG signal components associated with fatigue.

## 3. Discussion

Wearable biomechanical devices have experienced rapid advancements in the realm of sports fatigue monitoring, with IMU, IPS, and sEMG systems representing the three most prominent technologies. A total of 31 studies were included in this review (Table 1), comprising 14 IMU-based, 7 IPS-based, and 10 sEMG-based investigations. With respect to research objectives, only a small subset of studies quantitatively examined biomechanical changes before and after fatigue, whereas the majority focused primarily on evaluating the accuracy of fatigue recognition or classification models. Nearly half of the included studies relied solely on subjective RPE scores or similar rating scales as the criterion for defining fatigue. A smaller proportion combined subjective evaluations with objective indicators [21,36,38]. In contrast, sEMG-based studies more frequently adopted objective fatigue criteria, such as changes in EMG spectral metrics (e.g., MF/MPF/MDF), reductions in muscle fiber conduction velocity (MFCV), or task failure. In addition, only seven studies explicitly described their calibration procedures. Regarding model generalizability, only nine studies reported cross-subject validation results, most of which were based on IMU systems, indicating that cross-participant evaluation has not yet become a routine methodological component in this field. These discrepancies collectively demonstrate substantial heterogeneity in fatigue definitions and labeling standards across existing studies, which not only limits the comparability of measurement baselines but also constrains the accumulation of a coherent methodological framework. It is also notable that current studies rarely provide explicit thresholds for model performance. Instead, reported accuracy is often interpreted in relative terms, with higher values being described as “good” or “excellent”, typically in comparison with baseline models or prior work rather than with respect to task-specific benchmarks or application requirements.

Existing studies indicate that IMU measurements correlate well and agree consistently with traditional fatigue indicators such as the Rating of Perceived Exertion, thereby supporting the utility of IMUs in fatigue monitoring. As research advances, IMUs have demonstrated strong capabilities in fatigue detection during high-load tasks such as running and construction work. However, most IMU studies involve small sample sizes and are focused on healthy athletes and construction workers, which limits the generalizability of the findings to broader populations. Notably, the reported changes in knee joint angles after fatigue vary across studies. Individuals may adopt different compensatory strategies under various motor tasks, resulting in adaptive kinematic changes of the knee in varying directions. On the other hand, discrepancies in sensor placement and joint angle estimation methods may also contribute to inconsistent results [99]. The accuracy of IMU-based orientation and joint angle estimation is highly dependent on sensor placement and its alignment with the anatomical coordinate system, with any mounting deviation or micro-movement introducing systematic errors. Variations in the orientation estimation algorithms used across studies, such as complementary filters and EKF, differ in their capacity to suppress drift and handle noise, further contributing to inconsistencies in post-fatigue joint angle outcomes. Therefore, future research is needed to utilize external gold standards for cross-validation to distinguish between task effects and algorithmic differences, thus more accurately elucidating the actual impact of fatigue on knee joint kinematics. Moreover, while combining multiple sensors can enhance fatigue detection accuracy, it compromises wearability and increases workflow complexity. Future research should focus on optimizing sensor configuration strategies. Developing modular IMU arrays with self-calibration capabilities could help strike an optimal balance between detection performance and wearing comfort [100].

IPS systems hold unique advantages in revealing how fatigue affects gait stability and lower limb loading strategies. With the integration of intelligent algorithms, its fatigue recognition capability has expanded from single movement modes to multi-posture scenarios, and further extended to applications such as load management and injury risk warning. However, current IPS-based applications still face several key challenges. First, because insoles do not fully conform to the foot and are usually fixed in specific positions within the shoe, inevitable relative slippage during gait can compromise the accuracy of gait localization. Therefore, improving foot-position estimation during monitoring is critical [101]. Artificial intelligence (AI) has the potential to detect and correct such slippage by identifying abnormal pressure-distribution patterns, and may be deeply integrated with IPS for real-time error compensation in the future. Additionally, inter-individual variations in foot anatomy and gait mechanics can also affect data quality. To address this, future research should explore calibration strategies that integrate personalized plantar models with high-density sensor arrays to minimize system errors caused by anatomical and motion variability [83].

EMG reflects muscle activity and functional status, making it a precise and convenient technology for fatigue monitoring. Currently, wearable sEMG systems are primarily validated in short-term laboratory settings, with most research focusing on lower limb muscle fatigue during running, strength training, and cycling. To the need for real-time monitoring, commonly used features such as RMS and MDF, low-dimensional, are widely adopted for fatigue prediction. While these features are easy to deploy, they are highly sensitive to motion artifacts, electrode displacement, and impedance changes caused by sweat, which limits their stability for long-term monitoring [50]. To address these limitations, some studies have introduced machine learning and deep learning methods to extract high-dimensional latent features, yielding partial performance improvement. However, current models remain constrained by small datasets, and their generalizability across individuals and scenarios requires further enhancement. Recent advances in electrospun nanofiber-based composites have enabled a series of soft, skin-conformal electrodes for EMG and other electrophysiological monitoring, which can minimize sweat-induced impedance drift and motion-related artifacts [57,102]. Therefore, incorporating such emerging breathable electrode technologies into wearable fatigue-assessment systems may be a promising pathway for enhancing the stability of sEMG-based fatigue monitoring. Future work should focus on combining breathable electrode materials with robust signal processing strategies, and on constructing large-scale, multi-environment datasets to enhance the reliability of sEMG-based fatigue detection.

Moreover, the trend toward miniaturization and wireless design imposes increasingly stringent requirements on energy management. Most current wearable devices still rely on batteries with limited capacity, which are insufficient to support continuous monitoring in real-world conditions. Therefore, targeted innovations in energy supply and power consumption control are essential. The development of self-powered technologies provides a promising pathway toward enabling long-term monitoring. Among these, energy harvesting strategies based on triboelectric nanogenerators can convert human mechanical energy into electricity, exhibiting high sensitivity and strong environmental adaptability, thereby potentially reducing reliance on traditional batteries [103]. In addition, flexible biofuel cells powered by lactate in sweat exhibit excellent biocompatibility and show considerable potential for powering low-power sensors, enabling long-term monitoring [104,105].

In contrast to single-modality approaches, multi-modal fusion techniques provide a more comprehensive representation of fatigue by capturing its multidimensional physiological and biomechanical signatures. Current trends indicate that wearable biomechanical devices are evolving from single-modality monitoring toward multi-modal information fusion. Most existing multimodal fatigue-monitoring systems rely on early feature-level fusion, where handcrafted features from heterogeneous sensors (e.g., IMU and sEMG) are concatenated prior to model training [23,31,75]. Only one has moved toward attention-based fusion, enabling dynamic reweighting of multimodal feature streams according to their task relevance [32]. Limited forms of late fusion have also been reported in early occupational safety systems, where fatigue-related decisions are integrated at the control layer to trigger alarms or equipment shutdown [21]. However, integrating data from different dimensions necessitates addressing technical issues such as signal synchronization and feature alignment. Without proper management of modality-specific noise and uncertainty, the fusion process may introduce additional sources of error. Consequently, researchers must implement rigorous calibration procedures and comprehensive cross-condition validation to ensure stable and reliable fatigue assessment results across varying conditions [30,106].

Beyond detecting fatigue states, an equally critical question concerns how such information supports intervention in real-world deployment. At present, most wearable biomechanical monitoring systems primarily focus on fatigue-state recognition and early warning. Only a limited number of studies have explored preliminary intervention strategies, such as vibration or color-coded alerts to raise users’ awareness of fatigue [21,31,35,37,43,47], or the use of assistive devices (e.g., exoskeletons) as indirect means of reducing muscular load [49]. Moreover, some studies have pointed out that the system holds potential for dynamically scheduling rest intervals or adjusting treatment plans based on the user’s fatigue level, and is expected to support the development of personalized training and rehabilitation programs in the future [32,107]. However, empirical evidence clarifying how fatigue-detection outputs can be systematically translated into concrete training adjustments or rest strategies remains notably insufficient.

Protecting user data privacy is essential, particularly when the monitoring process encompasses data collection, wireless transmission, cloud storage, and potential application sharing. As wearable systems move toward long-term, real-world deployment—particularly in occupational environments—issues such as data ownership, consent management, long-term compliance, and ethical oversight will become increasingly important.

## 4. Conclusions

This review highlights advancements in wearable devices designed for the biomechanical monitoring of exercise fatigue. Current evidence indicates that IMU, IPS, and sEMG systems facilitate portable, real-time, and noninvasive fatigue monitoring, demonstrating potential applications across various sports and occupational settings. The integration of machine learning and deep learning algorithms has markedly enhanced the accuracy of fatigue detection, as well as the efficiency of real-time monitoring and analysis. Nevertheless, despite these significant advancements, certain shortcomings remain in the research. Most studies involve small sample sizes and primarily examine athletes and construction workers. The substantial heterogeneity in fatigue definitions, calibration practices, and validation strategies observed across current studies represents a key bottleneck to improving methodological consistency and cross-study comparability in this field [108]. Additionally, various technical challenges persist across different devices: IMU must balance wearing comfort with performance monitoring; IPS is prone to sensor slippage, leading to positioning inaccuracies; and sEMG is vulnerable to noise and motion artifacts, which compromise signal stability. Furthermore, limitations in battery life continue to constrain the continuous monitoring capabilities of these devices. These limitations compromise signal fidelity and system robustness in prolonged monitoring scenarios, thereby reducing the consistency of real-time fatigue assessment. AI is expected to play a pivotal role in addressing these challenges by enabling adaptive feature extraction, intelligent drift compensation, and robust multimodal data fusion, thereby enhancing the long-term accuracy, stability, and user-friendliness of fatigue monitoring systems [109,110]. The incorporation of lightweight algorithms and edge-computing architectures further supports low-power, real-time processing, which is essential for improving system portability and user-friendliness [109,110]. Consequently, future work should emphasize large-scale validation across multiple centers and varied movement conditions, as well as the exploration of modular sensing structures, self-calibration strategies, and self-supply technologies. By fostering multi-modal data fusion and intelligent analytics, future systems could deliver more continuous, accurate, and efficient fatigue management, and injury prevention and health promotion can be achieved for both sports and occupational populations.

## Figures and Tables

**Table 1:** Summary of fatigue monitoring techniques using IMU, IPS, and EMG.

Authors	Research Purposes	Modality	Sample	Algorithm Types	Fatigue Classes	Ground-Truth Labels	Sensor Placement	Research Results
Strohrmann C et al. [20], 2012	Aims to monitor kinematics by extracting kinematic parameters from motion data collected using wearable sensors.	IMU (Multi-axis)	21 runners	A complementary filter approach (Supervised learning)	Binary classification	Borg CR10	Lower limb	VO increased significantly on the treadmill by 8.12% with fatigue. The beginner was exposed to higher impact accelerations than the advanced runner and the impact increased with fatigue. HL decreased significantly over time for all runners during treadmill and outdoor running. The maximum knee rotation velocity decreased for all runners.
Li et al. [21], 2014	Aims at reducing the risk of injury and thus increasing worker safety.	IMU (Multi-axis)	3 subjects	Acceleration variance analysis, FFT	Binary classification	Head posture	Head	By analyzing the acceleration data of the IMU sensor, fatigue and non-fatigue head movements can be effectively distinguished. A risk assessment model was developed by integrating IMU and EEG data to successfully assess the accident risk level (low, medium, and high) faced by workers.
Zhang J et al. [22], 2014	Aims to monitor the kinematics of walking in unconstrained environments using an IMU situated at the sternum during fatigue and no-fatigue walking conditions.	IMU (Multi-axis)	17 subjects	SVM (Supervised learning)	Binary classification	Maximum voluntary isokinetic exertions	Lower limb, Trunk	60% of the baseline MVE is categorized as fatigued state. Heel contact velocity was significantly increased in post-fatigue walking trials. Linear (accuracy 97%) and radial basis function (accuracy 96%) kernels performed equally well in intra-individual fatigue/no-fatigue classifications. SVM achieved about 90% inter-subject fatigue classification accuracy with general features for identifying fatigue among participants.
Op De Beéck T et al. [23], 2018	Aims to explore whether machine learning can be used to predict the RPE from inertial sensor data of individuals running outdoors.	IMU (Multi-axis)	29 runners	GBRT, ANN, EN (Supervised learning)	Ternary classification	Borg CR10 (0–10)	Upper limb, Lower limb	Three fatigue levels are established: hard, very hard, and extremely hard. GBRT achieves the best performance using data from the wrist; LASSO and EN perform better on the arm and tibia. Fusing the data from multiple sensor locations cannot improve predictive performance. Normalizing the RPE clearly improves the MAEs in all runners.
Guan XL et al. [24], 2021	Aims to develop wearable exercise fatigue detection technology to estimate the human body’s exercise fatigue state using real-time monitoring of inertial sensor signal of the human body.	IMU	14 subjects	Bi-LSTM (Supervised learning)	Ternary classification	Borg RPE 6–20	Trunk	Bi-LSTM model achieved classification accuracy of 80.55% for three levels of exercise fatigue (relaxed, moderate fatigue, and severe fatigue).
Elshafe M et al. [25], 2021	Aims to evaluate the reliability and accuracy of a machine-learning model for monitoring bicep muscle fatigue during exercise.	IMU	20 subjects	FNN (Supervised learning)	Binary classification	Borg RPE 6–20	Upper limb	Using a two-layer FNN achieves high performance for subject-specificity and cross-specificity in terms of precision (95%, 87%), recall (93%, 89%), accuracy (94%, 88%), and Fi-measure (94%, 88%) respectively.
Marotta L et al. [26], 2021	Aims to assess whether IMU can be used to distinguish among fatigue levels during an outdoor run with a machine-learning classification algorithm trained on IMU-derived biomechanical features.	IMU	8 runners	RF (Supervised learning)	Ternary classification	Borg RPE 6–20	Lower limb, Trunk	Three fatigue levels are established: no fatigue, mild fatigue, and severe fatigue. The optimal combination of lower limb and trunk IMU position is proposed. In each IMU configuration, the change in joint angle is the most significant. The left tibia was the recurrent sensor location. The accuracy of fatigue identification using RF ranged between 76.1% (single left tibia location) and 90.5% (all IMU locations).
Jiang YR et al. [27], 2022	Aims to estimate the fatigue levels based on the forecasted motion signal with a new novel time series approach.	IMU	12 subjects	Transformer-based Spatio-Temporal Attention Model (Unsupervised learning)	Classification of continuous variables	Fatigue scale 1–10	Upper limb, Lower limb	Compared with current forecasting models, the proposed model achieved the best results with an average of 0.18 of RMSE and 95% correlation coefficient between true IMU signals and forecasted IMU signals. The proposed network can accurately forecast a time horizon of up to 80 time_steps for motion signal forecasting and fatigue classification. The proposed mode achieved an accuracy of 83.5% and Pearson correlation coefficient of 92% between the estimated fatigue level and self-rated fatigue level.
Truppa L et al. [28], 2022	Aims to study variations in human kinematic parameters after a football-specific fatigue protocol.	IMU	16 football referees	Propose the “Fatigue Approximation Index (FAI)” to quantify fatigue levels based on changes in power parameters	Binary classification	Fatigue approximation index	Lower limb, Trunk	A fatigue protocol for performance assessment and fatigue detection/quantification based on four wearable sensors and a simple but reliable CMJ exercise was developed. 19 kinematic parameters were identified as suitable indicators for fatigue detection, including displacement parameters, power and energy parameters, velocity parameters, time parameters, and articular joint kinematic parameters.
Wang et al. [29], 2022	Aims to develop a low-cost, non-invasive fatigue assessment method to reduce the risk of running-related injuries.	IMU	19 runners	RF, SVM (Supervised learning)	Ternary classification	Borg RPE 6–20	Lower limb, Trunk	Three fatigue levels are established: pre fatigue, mid fatigue, and post fatigue. The RF model performs better than the SVM model at all IMU positions. The accuracy of the RF model using a single tibia IMU reached 87.21%, while the combination of tibia and thigh IMU had the highest accuracy of 91.10%.
Chang PF et al. [30], 2023	Aims to establish a modeling method based on IMU-based subdivision action mode evaluation, and to explore the classification performance of different deep learning models in predicting running fatigue stage.	IMU	19 runners	LSTM, CNN (Unsupervised learning)	Ternary classification	Borg RPE 6–20	Lower limb	The RPE value increases with the increase of exercise time, and the difference between the three stages is significant. The CNN and LSTM models can effectively complete the classification of fatigue IMU data. The hybrid model of CNN and LSTM is superior to the independent model, and the accuracy is 99.6%.
Bangaru S et al. [31], 2025	Aims to develop a program that automatically predicts oxygen uptake, using workers’ forearm muscle activity and exercise data to build a fatigue prediction model.	IMU	10 subjects	Bi-LSTM (Supervised learning)	No classification	Maximal oxygen uptake	Upper limb	The predicted average oxygen uptake was 9.22 mL/kg/min, which was very close to the actual measured 9.18 mL/kg/min. The BiLSTM model shows the lowest error and highest correlation in each sensor combination. The performance of IMU + sEMG sensor combination is better than that of IMU and sEMG alone.
Hwang S et al. [32], 2025	Aims to develop a fatigue detection system suitable for daily walking.	IMU	35 subjects	Hybrid CNN-LSTM-Attention Model (Supervised learning)	Binary classification	Borg RPE 6–20	Lower limb	The proposed model can effectively identify the fatigue and non-fatigue states of walking. The combination of EMG and IMU signals is superior to a single mode, with an accuracy rate of 86.98% and the highest fatigue recall rate (87.94%).
Cao ZS et al. [33], 2021	Aims to design a human fatigue detection system based on plantar pressure signals.	IPS	10 subjects	RF, SVM, GBDT (Supervised learning)	Five-class classification	Maximal oxygen uptake	Plantar	The average proportion of pressure distribution in the eight plantar regions showed that the largest proportion was the heel, followed by the first metatarsal; the second was the third metatarsal, and the smallest proportion was the first toe. Five exercise fatigue levels were classified: normal state, 60%, 70%, 80%, and 90% of maximal oxygen uptake. The RF model had a prediction accuracy of 82.5% for fatigue, followed by GBDT and SVM.
Zhang GX et al. [34], 2021	Aims to explore the changes of asymmetry and variability of plantar pressure metrics in different plantar foot regions upon prolonged brisk walking on treadmills among the elderly.	IPS	15 subjects	Friedman test and Wilcoxon signed rank test	Ternary classification	Borg CR10 (0–10)	Plantar	Eleven participants (73.3%) reported a severe level of fatigue; three (20%) and one participant (6.7%) reported a moderate level of fatigue and mild fatigue, respectively. There are significant differences in contact time among three time conditions (1st min, 30th min, 60th min) The PP of the forefoot and arch of two feet showed significant differences among the three time conditions. For the ASI of peak plantar pressure between the non-dominant and dominant feet, the medial arch and lateral arch showed significant differences among three time conditions. For the average of MAD, medial arch, lateral arch, and medial heel in both non-dominant and dominant feet showed significant differences among three time conditions.
Antwi-Afari MF et al. [35], 2023	Aims to utilize a wearable insole device to identify and classify physical fatigue levels in construction workers.	IPS	10 subjects	KNN, SVM, ANN, DT, RF, (Supervised learning)	Four-class classification	Borg RPE 6–20	Plantar	Four classes of physical fatigue levels were identified: no-level, low-level, medium-level, and high-level. The RF algorithm achieved the best classification performance (86%), followed by the KNN, SVM, DT, and ANN algorithms. The precision, recall, specificity, and F1 score metrics of the RF algorithm obtained classification performance 52.63–82.62%, 52.63–84.32%, 89.60–92.33%, and 52.63–83.46%, respectively.
Buxton J et al. [36], 2023	Aims to determine the effect of fatigue on PP and SA at multiple gait speeds in young well-trained athletes.	IPS	16 runners	Linear regression and Wilcoxon signed rank test	Binary classification	Heart rate, Blood lactic acid	Plantar	Pre-fatigue PP significantly increased from walking to jogging and from jogging to running with no difference between running and sprinting. Post-fatigue PP for walking was less than jogging, running, and sprinting. Post-fatigue PP was significantly greater compared to pre-fatigue PP at all moving modes.
Tian Y et al. [37], 2024	Aims to propose an intelligent insole system based on deep learning for multi-functional foot health monitoring and management.	IPS	600 subjects	1D-CNN deep learning algorithm (Supervised learning)	Binary classification	Borg RPE 6–20	Plantar	The model successfully identified the fatigue state in three modes of walking, running and jumping, and the average classification accuracy was 95.3%. The results are highly consistent with the standard force platform.
Simon S et al. [38], 2024	Aims to evaluate fatigue, the effect of physical load on ergonomic risk score and plantar pressure in the actual working environment.	IPS	24 subjects	Repeated measures analysis of variance	Four-class classification	Borg CR10(0–10)	Plantar	Four classes of physical fatigue levels were identified: low-level, medium-level, high-level, and extreme-level. Participants’ levels of fatigue increased from mild to moderate at the pre-test to higher levels at the post-test. The results did not identify any significant differences in plantar pressure values in the pre- and post-test comparison, but the plantar pressure value of the right foot is higher than that of the left foot.
Lang S et al. [39], 2025	Aims to develop a machine learning model based on insole pressure signals for continuous and real-time estimation of vertical ground reaction force and tibial force during running.	IPS	9 subjects	Temporal Convolutional Network and Transformer modules, CNN, LSTM (Supervised learning)	No classification	MDF Borg RPE 6–20	Plantar	Fatigue changes were indirectly reflected by continuous monitoring of lower limb load through IPS. The model achieved a normalized root mean square error of 7.33% for vertical ground reaction force prediction and 10.64% for tibial force prediction, with performance superior to traditional baseline models such as CNN and LSTM.
Heaffey J et al. [40], 2015	Aims to track muscle fatigue by estimating MFCV by ASIC.	sEMG	20 subjects	The BLE System on Chip (Unsupervised learning)	Classification of continuous Variables	MFCV	Lower limb	The MFCV decreases when the muscles became fatigued. Estimation of the MFCV was found to have an error of 3.1%.
Kobayshi T et al. [41], 2017	1. Aims to develop a muscle fatigue detection system using capacitance coupling electrodes. 2. Aims to measure muscle fatigue of the anterior and posterior tibialis muscles.	sEMG	5 subjects	A feature analysis method based on the ratio of iEMG, MDF and LFC (Unsupervised learning)	Classification of continuous Variables	iEMG, LFC	Lower limb	The ratio of iEMG decreased after exercise. LFC increased after exercise and became wave slowing. The developed system could measure EMG signals because similar results were obtained compared with those measured using a conventional system.
Nourhan T et al. [42], 2017	Aims to predict and detect muscle fatigue when it occurs.	sEMG	3 subjects	ANN (Supervised learning)	Binary classification	RMS, MDF	Upper limb	The ANN was used to successfully classify the RMS and MDF into non-fatigue and fatigue classes, thus achieving the prediction of muscle fatigue. The overall confusion matrix shows 99.6% accuracy and 0.4% error.
Liu S. H et al. [43], 2019	Aims to develop a wearable EMG patch to detect real-time gastrocnemius muscle fatigue while exercising.	sEMG	20 subjects	FFT, EMD (Unsupervised learning)	Classification of continuous Variables	MF	Upper limb	The ARM Cortex-M4 processor could run the FFT and EMD algorithm to detect the MF values of the EMG signals. MF values have a significant shift towards a lower frequency during exercise. The real-time MF values measured using the EMG patch were very close to the off-line MF values measured with the computer system.
Khan T et al. [44], 2019	1. Aims to investigate whether machine learning method can predict fatigue in a running task. 2. Aims to develop a segmentation of fatigue levels.	sEMG	12 subjects	RF, CPS (Supervised learning)	Ternary classification	Blood lactic acid	Lower limb	A class of fatigue level was identified as aerobic, anaerobic, or recovery phase. The best fatigue predictions based on lactate were achieved using the sensors on right and left vastus medialis. The time-event features showed the highest model performance in the model. The recovery phase and aerobic phase could be discriminated significantly by using the right and left vastus lateralis features.
Yousif HA et al. [45], 2019	Aims to evaluate lower limb muscle fatigue during running based on the EMG signals.	sEMG	10 subjects	Linear regression analysis of time-domain and frequency-domain features	Classification of Continuous Variables	MNF, MDF	Lower limb	MNF and MDF characteristics were identified as muscle fatigue index. The first running strategy with the lowest MDF and MNF indices, was more effective in reducing muscle fatigue.
Yahao S et al. [46], 2022	Aims to propose a fatigue evaluation and detection system based on EMG frequency domain features and validate on both isometric muscle contraction fatigue and dynamic muscle fatigue.	sEMG	10 subjects	Power Spectral Density Analysis based on FFT	Classification of Continuous Variables	Unable to complete the action	Upper limb, Lower limb, Trunk	MPF is gradually decreasing with increasing experimental time, and MPF decreased by about 20% in all subjects’ muscles after fatigue occurred compared to the initial values. MPF can be used to detect isometric contraction muscle fatigue and dynamic muscle fatigue.
Gehlot A et al. [47], 2022	Aims to monitor muscle fatigue with wearable myoelectric devices based on the Internet of Things.	sEMG	6 subjects	Based on threshold rule	Ternary classification	RMS	Lower limb	The threshold value for three muscle fatigue conditions is determined as mean RMS value as >2 V: Extensive; 1–2 V: Moderate, and <1 V: relaxed.
Gong Q. B et al. [48], 2023	1. Aims to propose a strategy for feature selection and pattern identification and assess muscle strength and fatigue in real time. 2. Aims to develop a flexible wireless sEMG fatigue monitoring system.	sEMG	600 subjects	Linear regression analysis of time-domain and frequency-domain features	Classification of Continuous Variables	Task Failure	Upper limb	The magnitude of RMS, MAV, and VAR have a positive relationship. As the muscles contract, MNF and MDF continue to decrease; there is a linear relationship between these two frequency-domain features. RMS has the highest significance and can be used to assess muscle strength. The ratio of MNF features value of 45.8%, and the linear decrease in frequency with time, which can be used to assess muscle fatigue in real-time
Byeon H [49], 2024	Aims to propose a new method that combines wearable sensors and medical robots to evaluate the fatigue state of passive lower extremity exoskeleton users.	sEMG	18 subjects	Electromyographic fatigue threshold calculation, Knee joint motion variance analysis	Classification of continuous variables	EMG fatigue threshold	Lower limb	The exoskeleton effectively delays EMGFT with an average delay of 42.9%, especially in the skilled group and the strong group. The exoskeleton effectively reduced the variance of knee abduction angle and improved the overall stability by 75.8%.
Zhang G et al. [50], 2025	Aims to propose a MACNet for a three-level assessment of muscle fatigue based on sEMG.	sEMG	48 subjects	Multiple Attention and Convolution Network (Supervised learning)	Ternary classification	Borg RPE 6–20	Upper limb	The MACNet model successfully identified the three categories of muscle fatigue based on the subjective fatigue ratings (low fatigue, medium fatigue, high fatigue). The average classification F1-Score and accuracy of MACNet are 83.95% and 84.11% subject-wise and 82.83% and 82.43% cross-subject, respectively.

ANN—artificial neural network; ASI—asymmetry index; Bi-LSTM—bidirectional long and short-term memory neural network; CPS—change-point segmentation; DT—decision tree; EMGFT—electromyographic fatigue threshold; EMD—empirical mode decomposition; EN—elastic net regularization; FFT—fast Fourier transform; FNN—feedforward neural network; GBDT—gradient boosting decision; GBRT—gradient boosted regression tree; GLM—generalized linear model; HL—heel lift; iEMG—integrated electromyography; KNN—K-nearest neighbor; LASSO—least absolute shrinkage and selection operator regularization; LFC—low-frequency component; LSTM—long short-term memory; MACNet—multiple attention and convolution network; MAE—mean absolute error; MDF—median frequency; MF—median frequency; MNF—mean frequency; MRF—mean power spectrum frequency; MVE—maximum voluntary isokinetic exertion; RF—random forest; RMS—root-mean-square; SVM—support vector machine; VO—vertical oscillation.

## Data Availability

No new data were created or analyzed in this study.
